# Melatonin may be associated with changes in HUWE1, AIM2 and PANoptosis‐related proteins following myocardial ischaemia–reperfusion in male rats

**DOI:** 10.1113/EP094192

**Published:** 2026-11-11

**Authors:** Engin Sahna, Gulnur Aslan, Muge Demir Boler, Zeynep Ozdemir, Sumeyye Keskin, Besir Er, Kazim Sahin

**Affiliations:** ^1^ Department of Pharmacology, Faculty of Medicine Dokuz Eylul University Izmir Turkey; ^2^ Department of Pharmacology, Faculty of Pharmacy Firat University Elazig Turkey; ^3^ Elazig Provincial Health Directorate Elazig Turkey; ^4^ Department of Pharmacology, Faculty of Medicine Uludag University Bursa Turkey; ^5^ Department of Medical Services and Techniques, Vocational School of Health Services Firat University Elazig Turkey; ^6^ Department of Animal Nutrition and Nutritional Disorders, Faculty of Veterinary Medicine Firat University Elazig Turkey

**Keywords:** AIM2, HUWE1, ischaemia–reperfusion, microRNA, mitophagy, PANoptosis

## Abstract

Melatonin secreted by the pineal gland is a neuroendocrine hormone that directly scavenges free radicals and activates receptor‐mediated signalling pathways in the cardiovascular system. This antioxidant molecule plays critical roles in processes such as limiting ischaemia–reperfusion (IR) damage, preventing apoptosis, correcting endothelial dysfunction and preventing myocardial remodelling. However, its role in multiple programmed cell death processes induced by IR remains uncertain. Understanding the role of PANoptosis, one of these processes, in myocardial IR damage and the effect of melatonin could lead to innovative treatment strategies. In this study, the aim was to investigate the effects of melatonin on myocardial IR damage, focusing on the levels of the mitophagy mediator HECT, UBA, and WWE domain‐containing E3 ubiquitin ligase 1 (HUWE1), PANoptosis proteins absent in melanoma 2 (AIM2), receptor‐interacting protein kinase 3 (RIPK3), and gasdermin D, as well as apoptotic protease‐activating factor 1 (APAF1) levels, and the levels of microRNA (miR)‐136 and ‐325 that may be associated with these proteins. Thirty minutes of ischaemia and 120 min of reperfusion were applied to the left coronary artery of the rats. Melatonin was administered at the beginning of reperfusion (10 mg/kg). Serum AIM2 and tissue HUWE1, RIPK3, gasdermin D and APAF1 levels were significantly increased whereas miR‐136 and miR‐325 levels were decreased; melatonin reversed these effects. The increase in serum AIM2 levels following IR damage and the decrease in these levels after melatonin administration suggest that serum AIM2 should be more thoroughly investigated as a potential biomarker for damage and treatment response.

## Abstract (highlights)

- **What is the main question of this study?**:
  Does melatonin alter the levels of PANoptosis‐related proteins and microRNA‐136 and ‐325 in myocardial ischaemia–reperfusion injury in rats?
- **What is the main finding and its significance?**:
  Melatonin reduced the infarct area; while decreasing the levels of HUWE1, AIM2, RIPK3, gasdermin D and APAF1, it increased the levels of microRNA‐136 and microRNA‐325. These findings provide the basis for investigating the relationships between melatonin's cardioprotective effects and the changes in PANoptosis‐related proteins and microRNAs.

## 1 INTRODUCTION

Ischaemic heart disease is among the leading causes of global mortality (WHO, 2025), and myocardial ischaemia–reperfusion (IR) injury is one of the critical pathophysiological processes determining the prognosis of this clinical condition (Aslan et al., 2020; Liu et al., 2022). Increased oxidative stress due to reperfusion leads to mitochondrial dysfunction in cardiomyocytes, triggering cell death and an inflammatory response (Chen & Fang, 2018). In this process, disruption of mitochondrial quality control mechanisms plays a pivotal role in exacerbating IR injury (Wang et al., 2025).

Mitophagy is a fundamental defence mechanism that enables the removal of injured mitochondria; however, under severe oxidative stress, mitophagic capacity is exceeded, leading to the accumulation of cytosolic danger signals and the activation of regulated cell death pathways (Nakahira et al., 2011; Vringer & Tait, 2023). In this context, the E3 ubiquitin protein ligase HECT, UBA, and WWE domain‐containing E3 ubiquitin ligase 1 (HUWE1) promotes activating molecule in beclin 1‐regulated autophagy protein 1 (AMBRA1)‐mediated mitophagy by regulating mitochondrial ubiquitination and AMBRA1 activation (Di Rita et al., 2018). Additionally, it has been shown that silencing HUWE1 reduces apoptosis in cortical neurons exposed to oxygen–glucose deprivation and reperfusion (He et al., 2019). These findings provide a rationale for investigating whether HUWE1 is associated with mitochondrial stress‐induced cell death responses in myocardial IR injury. Beyond mitochondrial quality control, HUWE1 directly interacts with absent in melanoma 2 (AIM2), mediating its polyubiquitination, thus promoting inflammasome assembly and caspase‐1 activation (Guo et al., 2020). This information suggests that the levels of HUWE1 and AIM2 are important in myocardial IR injury. Additionally, the role of HUWE1 in IR injury is unknown.

In recent years, it has been reported that cells when under pathological stress can activate multiple programmed cell death mechanisms simultaneously rather than a single death pathway, and this integrated response has been described as PANoptosis (Sun et al., 2024). PANoptosis is an inflammation‐related cell death programme that involves the coordinated activation of apoptosis, pyroptosis and necroptosis and is regulated by multiprotein complexes called PANoptosomes (Song et al., 2025). Although the role of PANoptosis in myocardial IR injury has recently been established, how this process relates to mitochondrial stress and mitophagy dysfunction remains unclear.

AIM2 is a sensor component of the PANoptosome and a mediator that recognizes cytoplasmic double‐stranded DNA (Guo et al., 2020). In cellular stress situations, sensors such as AIM2 activate the effector protein receptor‐interacting protein kinase‐3 (RIPK3) and the pore‐forming gasdermin D, thereby facilitating the integration of apoptotic, necroptotic and pyroptotic pathways to assemble the PANoptosome (Upmanyu & Upadhyay, 2025). Therefore, the regulation of AIM2 by HUWE1 provides a potential link to a broader network of inflammatory cell death. However, it has not yet been determined whether this connection operates in myocardial IR injury. RIPK3 and the pore‐forming protein gasdermin D participate in necroptotic and pyroptotic signalling, respectively (Chen et al., 2020). It has also been reported that apoptotic protease‐activating factor 1 (APAF1), a key regulator of mitochondrial apoptotic signalling, may interact with this network (Kang et al., 2025). However, studies evaluating these molecules together within the same pathophysiological mechanism in myocardial IR injury are quite limited.

Melatonin is an endogenous hormone that exhibits cardioprotective effects against myocardial IR injury due to its strong antioxidant and mitochondrial‐protective properties (Pedriali et al., 2026). However, the relationship between the protective effects of melatonin and the PANoptotic cell death programme, which is thought to be activated by IR injury, has not yet been clarified. On the other hand, microRNAs (miRs) are known to regulate cell death pathways at the post‐transcriptional level (Wei et al., 2019), Specifically, miR‐136 directly targets APAF1 in aged mesenchymal stem cells (Zhang et al., 2020) whereas miR‐325‐3p targets RIPK3 and attenuates necroptosis in a mouse model of myocardial infarction (Zhang et al., 2019). Together, these observations support investigating the effects of melatonin on miR‐136 and miR‐325 expression alongside changes in cell death‐associated proteins during myocardial IR injury.

Based on these observations, we hypothesized that melatonin‐mediated cardioprotection would be accompanied by changes in HUWE1, AIM2, and proteins associated with apoptotic, pyroptotic and necroptotic signalling, as well as changes in the expression of miR‐136 and miR‐325. In this context, the study investigated the effects of melatonin administered at the onset of reperfusion on myocardial infarction size, cardiac HUWE1, RIPK3, gasdermin D and APAF1 levels, serum AIM2 concentrations, and cardiac miR‐136 and miR‐325 expression (Figure 1).

**FIGURE 1:**
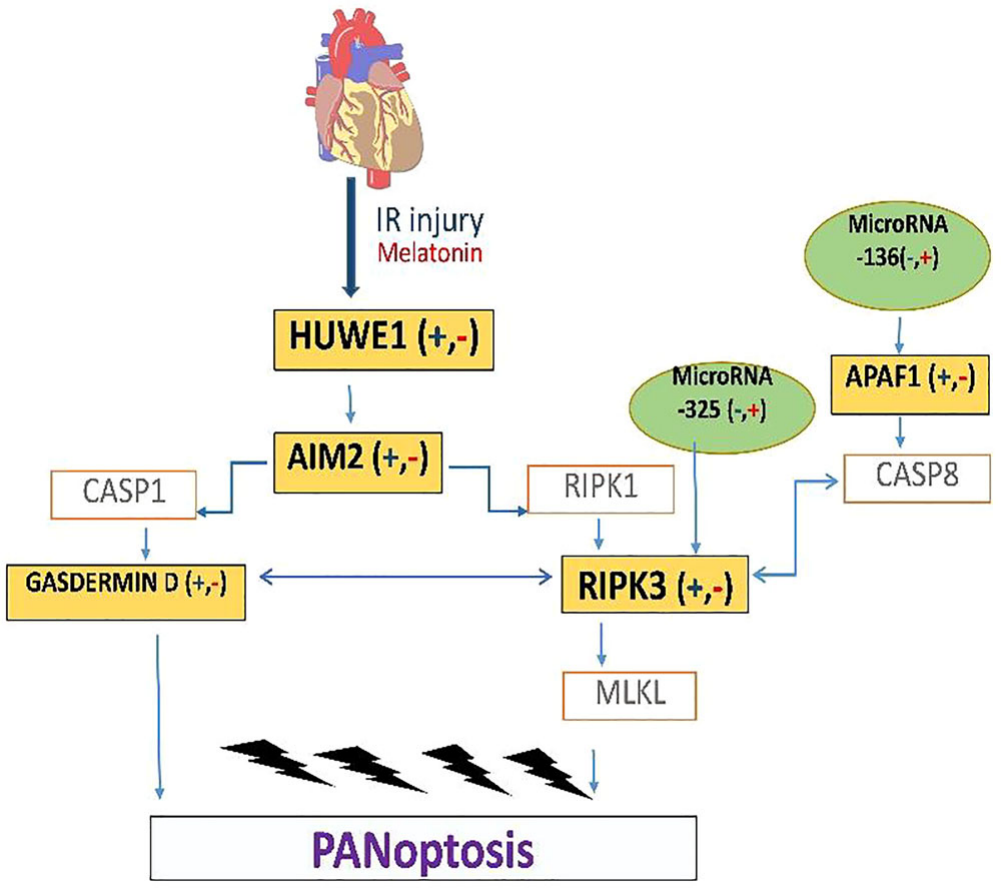
Graphical summary. Symbols indicate changes in the IR group versus Sham (first symbol) and in the IR+Mel group versus IR (second symbol): +, significant increase; −, significant decrease; ns, no statistically significant difference. AIM2, absent in melanoma 2; APAF1, apoptotic peptidase‐activating factor 1; CASP1/8, caspase‐1/8; HUWE1, HECT, UBA and WWE domain‐containing E3 ubiquitin protein ligase 1; IR, ischaemia–reperfusion; MLKL, mixed lineage kinase domain‐like protein; RIPK1/3, receptor‐interacting protein kinase 1/3.

## 2 METHODS

### 2.1 Ethical approval

All experimental procedures were approved by the Firat University Animal Experiments Local Ethics Committee (approval no: 2022/04) and were conducted in accordance with the animal welfare guidelines established by the institution and the National Institutes of Health *Guidelines for the Care and Use of Laboratory Animals*. The experiments complied with the animal experimentation policies of *Experimental Physiology*. The study is reported in accordance with the ARRIVE 2.0 guidelines.

### 2.2 Experimental groups

Fifty‐six adult male Sprague–Dawley rats (300 ± 50 g) were obtained from the Firat University Experimental Research Centre. The animals were housed under controlled laboratory conditions at 22 ± 2°C and 50–60% relative humidity, with a 12 h light/12 h dark cycle. They were fed commercial pellet feed and fresh drinking water and were not subjected to any special diet. Two independent cohorts were used for the evaluation of infarct size and biochemical analyses. The infarct size cohort consisted of 21 rats divided into the IR, IR+Mel and IR+Vehicle groups (7 rats in each group). The biochemical analysis cohort consisted of 35 rats divided into the Sham, Sham+Mel, Sham+Vehicle, IR and IR+Mel groups (*n* = 7 in each group). For the purpose of assessing infarct size, four consecutive tissue sections needed to be taken starting from the apex of the heart for TTC staining; as a result of this procedure, there was insufficient tissue remaining from the damaged myocardium for biochemical analyses. Therefore, separate animals were used for the two evaluations, and there were no common animals between the cohorts.

Considering the cardiovascular protective effects of oestrogen, male rats were chosen to limit the impact of fluctuations associated with the oestrous cycle in ovarian hormones. As a result, sex‐based differences in myocardial IR injury and the response to melatonin have not been evaluated.

### 2.3 IR procedure

Urethane (1.2 g/kg) was administered intraperitoneally as an anaesthetic. A tracheal cannula was placed for artificial respiration. The carotid artery was cannulated to monitor systemic blood pressure, and an ECG was recorded using a Biopac MP36. After a 1–1.5 cm skin incision on the left side of the chest, tissues and muscles were dissected, and a left thoracotomy was performed by cutting the fourth rib. Upon entering the thoracic cavity, positive pressure ventilation was initiated with a ventilation device to maintain respiration and $P_{CO_{2}}$, $P_{O_{2}}$ and pH values within normal ranges. The heart and pericardium were slowly detached and removed from the body with gentle pressure on the chest wall. A 10 mm round‐tipped needle was used to gently incorporate the myocardial tissue, and 6/0 silk thread was passed under the left coronary artery. The heart was then placed back into the chest cavity, and stabilization was allowed for 20 min. Follow‐up was performed according to the criteria specified in the Lambeth Conventions. Subsequently, the vessel was occluded by tightening the ends of a string passed beneath it. This experimental model had been successfully used before (Aslan et al., 2020). After 30 min of ischaemia and 120 min of cardiac reperfusion, the heart was rapidly removed and stored at −80°C for biochemical analysis.

### 2.4 Determination of the infarct size

Myocardial infarct size was determined using 2,3,5‐triphenyltetrazolium chloride (TTC; Sigma‐Aldrich, St Louis, MO, USA; cat. no. T8877; CAS No. 298‐96‐4; purity >98% by HPLC) staining. At the end of reperfusion, the excised rat heart was wrapped in aluminum foil and placed in the freezer for 12 h. The frozen hearts were sliced starting from the apex into 2 mm‐thick sections and incubated in a buffer solution containing 1% TTC (pH 7.4) at 37°C for 15–20 min. After incubation, the heart slices were placed between two glass plates. Live tissue and necrotic tissue (stained dark red and light brown, respectively) were marked on a transparent acetate sheet placed over the glass plate. The infarct size was measured using the ImageJ program and expressed as a percentage of the total heart tissue (Sahna et al., 2005).

Melatonin (Sigma‐Aldrich; cat. no. M5250; CAS no. 73‐31‐4; purity ≥98% by TLC) was dissolved in pure ethanol and diluted with saline to a final ethanol concentration of 0.01%. Melatonin was administered intraperitoneally at a dose of 10 mg/kg at the onset of reperfusion. Vehicle‐treated animals received an equivalent injection volume of saline containing 1% ethanol.

### 2.5 Western blot analysis

Relevant antibodies were used for western blot analysis of tissue HUWE1, APAF1, RIPK3 and gasdermin D levels. The samples were homogenized in phosphate‐buffered saline (PBS) containing the protease inhibitor mixture, and the total protein level was then calculated. Fifty micrograms per homogenate was mixed with the sample buffer and boiled for 5 min. Total proteins were separated via SDS‐PAGE and transferred onto nitrocellulose membranes using a semi‐dry transfer system. The membranes were washed in PBS and blocked with 1% BSA for 1 h before the primary antibody was applied. The membranes were subsequently incubated overnight at 4°C with the following primary antibodies: rabbit anti‐HUWE1 (1:1000; St John's Laboratory, cat. no. STJ11103418), rabbit anti‐RIPK3 (1:1000; Affinity Biosciences, Cincinnati, OH, USA, cat. no. A5431), rabbit anti‐APAF1 (1:1000; Affinity Biosciences, cat. no. AF01117), rabbit anti‐gasdermin D (1:1000; Affinity Biosciences, cat. no. A17308), and mouse anti‐β‐actin (1:3000; Santa Cruz Biotechnology, Dallas, TX, USA, cat. no. sc‐47778) (Orhan et al., 2016). For gasdermin D, the full‐length form (∼53 kDa) was exclusively evaluated to analyse protein expression levels. Immunoreactive bands were detected at their respective molecular weights: HUWE1 (∼437 kDa), APAF1 (∼130 kDa), RIPK3 (∼57 kDa), gasdermin D (∼53 kDa) and β‐actin (∼42 kDa). Following primary incubation, membranes were washed and incubated with a horseradish peroxidase (HRP)‐conjugated goat anti‐rabbit IgG (1:3000; Abcam, Cambridge, UK cat. no. ab97023) or goat anti‐mouse IgG (1:3000; Abcam, Cambridge, UK cat. no. ab97024) secondary antibody for 1 h at room temperature. Secondary antibodies were diluted in the same buffer containing 0.05% Tween‐20. Protein bands were visualized via 3,3′‐diaminobenzidine (DAB) staining. Densitometric analysis of the bands was performed using ImageJ.

### 2.6 Quantitative real‐time polymerase chain reaction analysis

Total miRNA was isolated using the mirVana™ miRNA Isolation Kit (Thermo Fisher Scientific, Waltham, MA, USA, cat. no. AM1561), according to the manufacturer's protocol. Reverse transcription (RT) was subsequently performed using the TaqMan™ MicroRNA Reverse Transcription Kit (Thermo Fisher Scientific, cat. no. 4366596). Since the detailed sequence information of the primers and probes is proprietary and not publicly disclosed by the manufacturer, commercially pre‐designed TaqMan miRNA assays were utilized. The target miRNAs and their specific product codes were as follows: miR‐136 (Ambion/Thermo Fisher Scientific, Product Code: P220526‐005 C10) and miR‐325 (Ambion/Thermo Fisher Scientific, Product Code: P230413‐004 G12). U6 small nuclear RNA (U6 snRNA) was used as the internal reference RNA for normalization. Quantitative PCR was conducted using TaqMan Universal PCR Master Mix on a Rotor‐Gene Q 96‐well real‐time PCR cycler (Qiagen, Hilden, Germany). The thermal cycling conditions were configured as follows: initial activation at 95°C for 10 min, followed by 40 cycles of denaturation at 95°C for 15 s, and annealing/extension at 60°C for 60 s. All reactions were performed in duplicate. The relative expression levels of mature miR‐136 and miR‐325 were calculated using the comparative threshold cycle $2^{-\Delta \Delta C_{T}}$ method.

### 2.7 ELISA analysis

Rat AIM2 levels in serum samples were quantified using a commercial sandwich enzyme‐linked immunosorbent assay (ELISA) kit (Shanghai YL Biotech Co., Ltd, Shanghai, China; cat. no. YLA1763RA), following the manufacturer's operational instructions. The kit protocol utilized a sandwich method with a detection range of 0.5–150 ng/mL and an assay sensitivity of 0.21 ng/mL. In brief, prepared serum samples, standards and biotin‐labelled antibodies along with streptavidin–HRP were added to the pre‐coated plate and incubated for 60 min at 37°C. After a five‐cycle washing phase to eliminate unbound components, Chromogen solutions A and B were added, and the plate was incubated for 10 min at 37°C for colour development. The enzymatic reaction was terminated by introducing the stop solution. The optical density (OD) was measured within 10 min at a wavelength of 450 nm using a microplate reader. Both intra‐assay and inter‐assay coefficients of variation (CV) were less than 8% and 10%, respectively. AIM2 concentrations were determined by interpolating the OD values onto the standard curve and final concentrations were expressed in ng/mL.

### 2.8 Statistics

In this study, data are presented as the mean ± standard deviation (SD). When *P *< 0.05, the difference between the groups was statistically significant. Normality was assessed using the Shapiro–Wilk test. Parametric tests were applied because the raw data met the assumptions of normality and homogeneity of variance. These tests were one‐way independent‐measures ANOVAs with Tukey's HSD *post hoc* tests. GraphPad Prism 10 (GraphPad Software, Boston, MA, USA) was used to draw the graphs.

## 3 RESULTS

IR‐induced myocardial infarct size (39.27 ± 3.48%) was significantly reduced with melatonin administration (32.83 ± 2.74%). No significant change was found in infarct size (38.97 ± 2.69%) in the melatonin solvent groups compared to the control groups (Figure 2).

**FIGURE 2:**
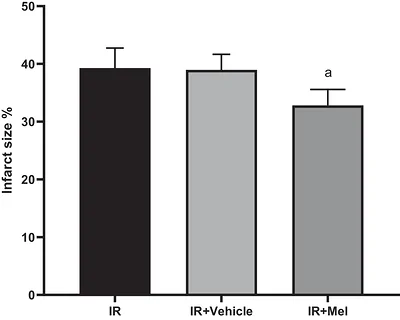
Effect of melatonin administration on infarct size. ^a^Significant difference compared to ischaemia–reperfusion, *P* = 0.021, *n* = 7. IR, ischaemia–reperfusion; Mel, melatonin (10 mg/kg).

Levels of AIM2, which acts as a sensor protein in panoptosome formation, were analysed by ELISA. Serum AIM2 levels were significantly higher in the IR group (5.12 ± 0.61 ng/ml) than in the Sham group (1.84 ± 0.14 ng/ml). In the IR+Mel group, a significant decrease was observed compared to the IR group (3.85 ± 0.34 ng/ml) (*P* = 0.008). In the Sham+Mel and Sham+Vehicle groups, the values were as follows: 1.81 ± 0.89; 1.78 ± 0.20 ng/ml (Figure 3).

**FIGURE 3:**
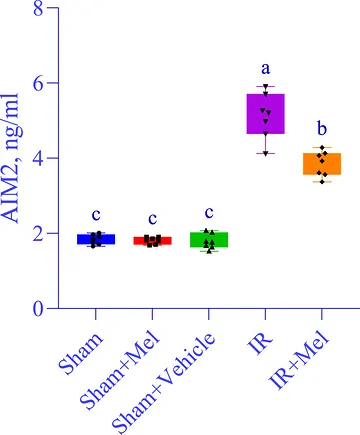
The changes of the serum AIM2 levels across experimental groups. Individual data points for each animal are displayed as black diamonds within the boxes (*n* = 7 per group). In the box‐and‐whisker plots, the horizontal line within each box represents the median, the box boundaries indicate the 25th and 75th percentiles, and the whiskers extend to the minimum and maximum values. Statistical analyses were performed using one‐way ANOVA followed by Tukey's *post hoc* test for multiple comparisons. Groups sharing the same superscript letters (a–c) are not significantly different from each other, whereas groups with different letters differ significantly. AIM2, absent in melanoma 2; Sham, sham‐operated control group; Sham‐Mel, sham + melatonin treated group; Sham‐Vehicle, sham + carrier solution control group; IR, ischaemia–reperfusion group; IR+Mel, ischaemia–reperfusion + melatonin treated group.

Expression levels ofAPAF1, another protein associated with PANoptosis, showed a significant increase (164.71 ± 4.3%) in the myocardial IR group compared to the Sham group (100 ± 2.26%); furthermore, a significant decrease (133.8 ± 3.32%) was recorded in the IR+Mel group compared to the IR group (*P* < 0.0001). In the Sham+Mel group, this value was 98.91 ± 4.61, while in the Sham+Vehicle group, the value was 93.72 ± 4.18 (Figure 4a).

**FIGURE 4:**
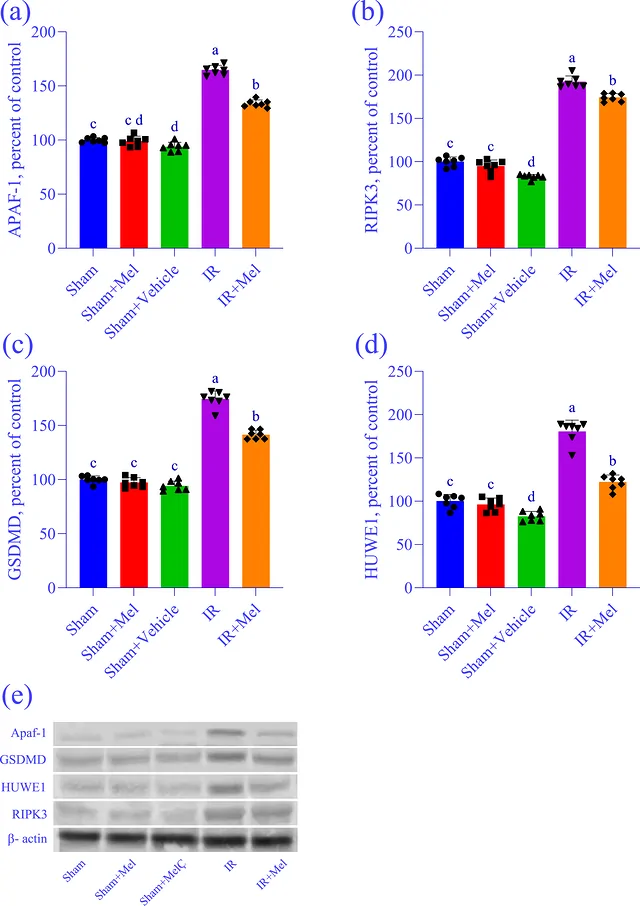
The changes of the protein expression of APAF1 (a), RIPK3 (b), GSDMD (c), and HUWE1 (d), alongside a representative Western blot image (e). Individual data points for each animal are represented as black dots within the bars (n:7 per group). Densitometric data are expressed as mean ± SD and presented as a percentage of the control group. Statistical analyses were performed using one‐way ANOVA followed by Tukey's *post hoc* test for multiple comparisons. Groups sharing the same superscript letters (a–d) are not significantly different from each other, whereas groups with different letters differ significantly. Panel E shows a representative image of the target proteins and the β‐actin loading control, which is identical for all blots as the same biological samples were used across all panels. GSDMD, gasdermin D; Sham, sham‐operated control group; Sham‐Mel, sham + melatonin treated group; Sham‐Vehicle, sham + carrier solution control group; IR, ischaemia–reperfusion group; IR+Mel, ischaemia–reperfusion + melatonin treated group.

When changes in the expression levels of RIPK3, one of the effector proteins in the PANoptosis process, were evaluated, a significant increase was recorded in the IR group (192.4 ± 6.68%) compared to the Sham group (100 ± 5.52%). The levels of this protein were significantly decreased in the IR+Mel group (174.2 ± 4.5%) compared to the IR group (*P* < 0.0001). In the Sham+Mel group, this value was 94.94 ± 7.13, while in the Sham+Vehicle group, the value was 82.31 ± 2.80 (Figure 4b).

Expression levels of gasdermin D, one of the pore‐forming proteins, were significantly increased in the IR group (174.5 ± 7.51%) compared to the Sham group (100 ± 3.33%). Levels of this protein were significantly decreased in the melatonin group (141.5 ± 4.17%) (*P* < 0.0001). In the Sham+Mel group, the value was 97.19 ± 4.50, while in the Sham+Vehicle group, the value was 94.09 ± 4.49 (Figure 4c).

Expression levels of HUWE1, a ubiquitin ligase involved in mitophagy, showed a significant increase (180.5 ± 13.07%) in the myocardial IR group compared to the Sham group (100 ± 7.80%); furthermore, a significant decrease (122.1 ± 8.14%) was recorded in the IR+Mel group compared to the IR group (*P* < 0.0001). In the Sham+Mel and Sham+Vehicle groups, the values ​​were 96.22 ± 7.45 and 82.5 ± 5.77%, respectively (Figure 4d).

miR‐136 mRNA expression levels were significantly decreased in the IR group (0.36 ± 0.002) compared to the control group (1.000 ± 0.017). A significant increase was recorded in the IR+Mel group (0.52 ± 0.003) compared to the IR group (*P* < 0.0001) (Figure 5a). miR‐325 levels were significantly lower in the IR group (0.245 ± 0.005) than in the control group (1.000 ± 0.021). However, miR‐325 levels were significantly increased in the IR+Mel group (0.56 ± 0.02) compared to the IR group (*P* < 0.0001). In the Sham+Mel and Sham+Vehicle groups, the values for miR‐136 were 1.29 ± 0.003 and 1.17 ± 0.001, respectively, and for miR‐325 1.21 ± 0.004 and 1.12 ± 0.003, respectively (Figure 5b).

**FIGURE 5:**
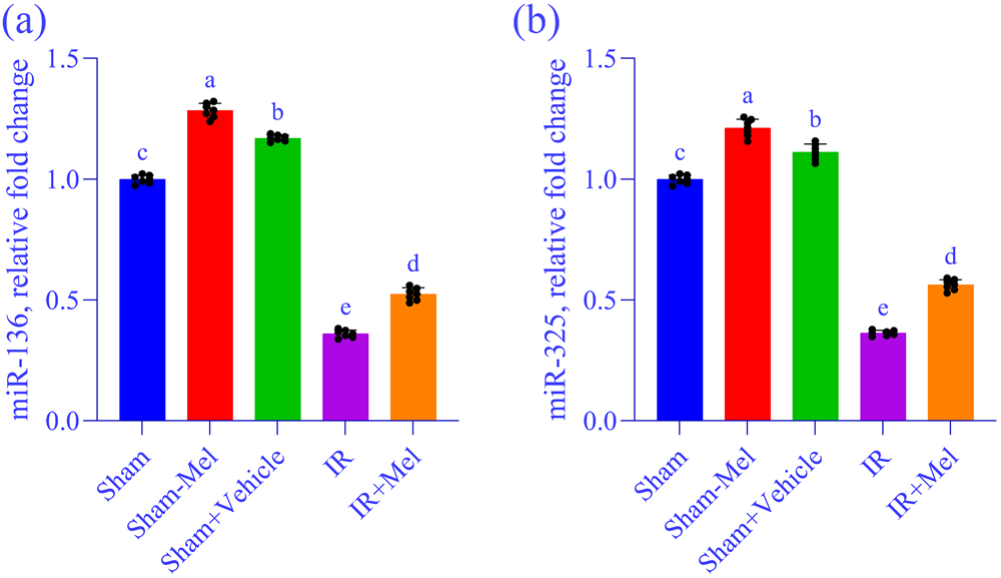
Effects of melatonin treatment on miR‐136 (a) and miR‐325 (b) expression levels. Individual data points for each animal are represented as black dots within the bars (*n* = 7 per group). Data are expressed as means ± SD. Statistical analyses were performed using one‐way ANOVA followed by Tukey's *post hoc* test for multiple comparisons. Groups sharing the same superscript letters (a–e) are not significantly different from each other, whereas groups with different letters differ significantly (*P* < 0.05). Sham, sham‐operated control group; Sham‐Mel, sham + melatonin treated group; Sham+Vehicle, sham + carrier solution control group; IR, ischaemia–reperfusion group; IR+Mel: ischaemia–reperfusion + melatonin treated group.

## 4 DISCUSSION

In our study, the levels of the mitophagy mediator HUWE1, the sensor protein AIM2, and the proteins RIPK3, gasdermin D and APAF1 involved in PANoptosis increased following IR injury, whereas melatonin administration significantly decreased their levels. miR‐136 and miR‐325 levels significantly decreased with IR injury; melatonin reversed all these effects, demonstrating that it can activate multiple programmed cell death mechanisms via the HUWE1–AIM2–PANoptosis axis by exceeding mitophagy capacity.

Cardiomyocytes obtain the energy they need for metabolism and contraction largely from mitochondria. When IR‐induced mitochondrial dysfunction reaches a certain threshold, mitochondrial and cell membranes are injured, their contents leak out of the cell, and an innate immune response is triggered (Liu et al., 2022). When dynamic mechanisms cannot repair mitochondrial damage, mitophagy begins, leading to the selective destruction of the organelle. HUWE1 induces mitochondrial clearance by ubiquitinating proteins involved in mitophagy (Di Rita et al., 2018). Our study showed an increase in HUWE1 levels with IR injury. Similarly, silencing HUWE1 was observed to reduce apoptosis in cortical neurons exposed to oxygen–glucose deprivation and reperfusion (He et al., 2019). Furthermore, HUWE1‐mediated AIM2 ubiquitination is necessary for inflammasome formation and caspase‐1 activation (Guo et al., 2020). Our study suggests that IR injury and the resulting increase in HUWE1 levels may have facilitated AIM2‐mediated PANoptosome formation and PANoptosis.

AIM2 is one of the sensor proteins involved in PANoptosome formation. It plays a critical role in inflammasome assembly and signal transduction by transmitting signals to effector proteins, such as caspase‐1 and RIPK1, through protein–protein interactions (Wu et al., 2025). Caspase‐1 activates caspase‐3 and caspase‐8 via gasdermin D to form pores in the cell membrane; RIPK1, on the other hand, activates RIPK3, leading to RIPK3‐mediated mixed lineage kinase domain‐like protein (MLKL) phosphorylation (Upmanyu & Upadhyay, 2025). RIPK3 interacts with caspase‐1 and caspase‐8, thereby mediating PANoptosis (Su et al., 2025). Ultimately, programmed cell death modes such as pyroptosis, apoptosis, and necroptosis are induced simultaneously, and inflammatory cytokine release is observed. It has also been shown that RIPK3 and gasdermin D collaborate to enhance inflammatory signalling and tissue injury (Chen et al., 2020)

Gasdermin D is the most highly expressed member of the gasdermin family in cardiac tissue (Broz et al., 2020). Gasdermin D deficiency has been reported to lead to a tendency towards apoptosis in cell fate (Zhou et al., 2024). Apoptosis is regulated by the apoptosome complex, exemplified by the detection of mitochondrially derived cytochrome *c* by APAF1 and its interaction with caspases (Li et al., 1997). APAF1 has been identified as a gene associated with PANoptosis, with potential diagnostic and therapeutic implications (Kang et al., 2025). Under inhibition of caspase 8 activity, PANoptosis is mediated by the interaction between RIPK3 and RIPK1 (Song et al., 2025; Vanden Berghe et al., 2016). In our study, AIM2 levels were significantly increased in IR injury. This increase suggested that it might have triggered the later stages of the PANoptosis process by inducing RIPK1‐mediated activation of RIPK3 and caspase‐1‐mediated increase in gasdermin D. Interpreting our findings with current literature, it is possible that, in accordance with the fate of PANoptosis, the increase in levels of AIM2, which acts as a sensor in the formation of the PANoptosome, triggers the later stages with IR injury, increasing the levels of the effector protein RIPK3 and the pore‐forming gasdermin D, thus initiating the PANoptotic process. Melatonin administration significantly reduced the levels of all these proteins, indicating significant protection by modulating them at every stage of the PANoptosis formation mechanism. Similarly, melatonin has been shown to protect by both reducing gasdermin D and caspase‐1 levels in ocular hypertensive mice (Ye et al., 2022) and improving PANoptosis parameters in spinal cord ischaemia (Xie et al., 2024).

MicroRNAs, which regulate target gene expression at the post‐transcriptional level, play a critical role in the pathophysiology of ischaemic injury by modulating oxidative stress and various cellular death pathways. In our study, miR‐136 and miR‐325 levels were significantly decreased with IR injury; melatonin reversed all these effects. Similarly, overexpression of miR‐325‐3p reduced damage in heart tissue, decreased infarct size, and downregulated the expression of necrosis‐associated proteins RIPK1, RIPK3, and p‐MLKL (Zhang et al., 2019). In our study, the decrease in RIPK3 levels due to melatonin treatment may have been mediated by melatonin's effect on miR‐325. miR‐136 is a negatively regulated downstream gene that directly targets the 3'UTR of APAF1 (Zhang et al., 2020). Another study has shown that miR‐136‐5p, via the ataxin‐1‐like–capicua axis, inhibits pyroptosis and protects cardiomyocytes from coronary microembolization (Cai et al., 2022). The significant changes in miR levels that melatonin induces may have contributed to the downregulation of PANoptosis by affecting APAF1 and caspase receptors.

The statistically significant differences observed between the Sham and Sham+Vehicle groups in certain parameters (particularly APAF1, HUWE1 and RIPK3) may be attributed to the fact that the vehicle solution is not entirely biologically inert. In this study, the low concentration of ethanol (1%) used in the preparation of the melatonin solution is in the literature generally considered to have minimal biological effects; however, it may exert mild modulatory effects, particularly on mitochondrial function, oxidative stress balance and cellular stress responses. This condition may have led to limited alterations in apoptotic (APAF1), necroptotic (RIPK3) and mitophagy‐related (HUWE1) pathways by inducing a low‐level stress response under basal conditions. However, it should be noted that these changes were limited to sham conditions and remained considerably low when compared to the pronounced increases observed in the IR model. Indeed, the elevations in these parameters in the IR group were substantially higher than those in the Sham and Sham+Vehicle groups, and melatonin administration significantly suppressed this pathological increase. Therefore, the differences between the Sham and Sham+Vehicle groups do not affect the main biological conclusions of the experimental model and suggest that the vehicle effect is limited and represents a secondary phenomenon associated with a systemic stress response.

In conclusion, melatonin administered at the onset of reperfusion reduced myocardial infarct size in male rats. This protective effect was accompanied by lower cardiac HUWE1, RIPK3, gasdermin D and APAF1 levels, reduced serum AIM2 concentrations, and higher miR‐136 and miR‐325 expression compared with untreated IR animals. Further functional studies are required to determine whether these molecular changes contribute causally to the protective effects of melatonin. However, the increase in serum AIM2 levels and its suppression by melatonin suggest that AIM2 may serve as a non‐invasive biomarker for PANoptosis and treatment response following ischaemia–reperfusion injury.

### 4.1 Limitations

The main limitation of the study is that the HUWE1–AIM2–PANoptosis association has not been confirmed by genetic or pharmacological inhibition. However, the findings are consistent with the literature and provide a strong framework for further mechanistic studies.

## AUTHOR CONTRIBUTIONS

Gulnur Aslan: Designing the study, analysing the data, and writing the article. Zeynep Ozdemir: Conducting the experiments and obtaining the data. Besir Er: Conducting the experiments and obtaining the data. Engin Sahna: Designing the study and critically reviewing the article in terms of significant intellectual content. Muge Demir Boler: Conducting the experiments and obtaining the data. Sumeyye Keskin: Conducting the experiments and obtaining the data. Kazim Sahin: Conducting the experiments and obtaining the data. All authors have approved the final version of the article and have agreed to be responsible for all aspects of the work, including its accuracy and integrity. All listed authors meet the authorship criteria, and everyone who meets these criteria is included in the author list. All experiments were conducted at Fırat University. Surgical procedures were performed in the Department of Pharmacology at the Faculty of Medicine, while biochemical analyses were conducted in the Department of Animal Nutrition at the Faculty of Veterinary Medicine. The authors declare that all data were produced in their own institutions, that no article production services (‘paper mill’) were utilized.

## CONFLICT OF INTEREST

The authors have no relevant financial or non‐financial interests to disclose.

## GENERATIVE AI STATEMENT

No artificial intelligence tools were used in the creation of text or figures. During manuscript preparation and revision, the authors used QuillBot Translator to translate author‐written text from Turkish into English. The authors take full responsibility for the accuracy and integrity of the final manuscript.

## Data Availability

The data supporting the findings of this study are available within the article and its Supporting Information. The original Western blot images are provided in the Supporting Information.
